# Effects of age on systemic inflammatory response syndrome and results of coronary bypass surgery

**DOI:** 10.5830/CVJA-2017-030

**Published:** 2018

**Authors:** Gokalp Orhan, Besir Yuksel, Yilik Levent, Gurbuz Ali, Karakas Yesilkaya Nihan, Iner Hasan, Durmaz Huseyin, Gokkurt Yasar, Lafci Banu, Bozok Sahin, Gokalp Gamze

**Affiliations:** Department of Cardiovascular Surgery, Faculty of Medicine, İzmir Katip Celebi University, İzmir, Turkey; Department of Cardiovascular Surgery, Faculty of Medicine, İzmir Katip Celebi University, İzmir, Turkey; Department of Cardiovascular Surgery, Faculty of Medicine, İzmir Katip Celebi University, İzmir, Turkey; Department of Cardiovascular Surgery, Faculty of Medicine, İzmir Katip Celebi University, İzmir, Turkey; Department of Cardiovascular Surgery, Ataturk Training and Research Hospital, İzmir Katip Celebi University, İzmir,Turkey; Department of Cardiovascular Surgery, Ataturk Training and Research Hospital, İzmir Katip Celebi University, İzmir,Turkey; Department of Cardiovascular Surgery, Ataturk Training and Research Hospital, İzmir Katip Celebi University, İzmir,Turkey; Department of Cardiovascular Surgery, Ataturk Training and Research Hospital, İzmir Katip Celebi University, İzmir,Turkey; Department of Cardiovascular Surgery, Ataturk Training and Research Hospital, İzmir Katip Celebi University, İzmir,Turkey; Department of Cardiovascular Surgery, Bahcesehir University, İstanbul, Turkey; Department of Pediatric Emergency, İzmir Tepecik Training and Research Hospital, İzmir, Turkey

**Keywords:** systemic, inflammation, coronary, bypass

## Abstract

**Background:**

Coronary artery bypass (CAB) surgery triggerssystemic inflammatory response syndrome (SIRS) via severalmechanisms. Moreover, age is directly correlated with SIRS.We evaluated the effect of age on SIRS and postoperativeoutcome after CAB surgery.

**Methods:**

We retrospectively reviewed the records of 229 patients who had undergone CAB surgery. The patients were divided into three groups according to age: group 1, < 40 years (n = 61); group 2, 40–75 years (n = 83); and group 3, > 75 years old (n = 85). Pre- and peri-operative data were assessed in all patients. SIRS was diagnosed according to the criteria established by Boehme.

**Results:**

The average pre-operative EuroSCORE value in group 3 was higher than in the other groups and body surface areas were significantly lower in group 3 than in the other groups (p < 0.05). The postoperative SIRS rates were 68.9% in group 1, 84.3% in group 2 and 91.8% in group 3 (group 1 vs group 3; p < 0.05). Mortality rates were not significantly different between the groups (p > 0.05). The predictive factors for SIRS were age, EuroSCORE rate, on-pump CAB surgery and intra-aortic balloon pump use.

**Conclusion:**

Age was an important risk factor for SIRS during the postoperative period after CAB.

## Background

Coronary artery bypass grafting (CABG) is the conventional treatment for coronary artery disease (CAD). Previously, CABG was primarily performed in patients between the ages of 60 and 75 years. However, because of increased life expectancy and the need to re-perform the procedure, CABG is now commonly performed in patients over 75 years of age.^1^–^3^ As a result of this age-related shift in CABG recipients, some postoperative outcome parameters have changed.

Systemic inflammatory response syndrome (SIRS) is an inflammatory process that can be triggered during openheart surgery. SIRS is produced by the release of several pro-inflammatory mediators and affects postoperative outcome after open-heart surgery.^3^,^4^ The recent marked increase in SIRS after CABG may be due to age-related changes in the immune system.^5^,^6^ We therefore investigated the correlation between age and SIRS after CABG.

## Methods

Ethics committee approval was obtained for the study. Patient medical records were obtained from the hospital automation system and archived files.

We retrospectively evaluated 229 patients who had undergoneCABG. The patients were divided into three groups accordingto age: group 1 patients were under 40 years old (n = 61), group 2 were 40–75 years (n = 83), and group 3 were over 75 years old(n = 85).

We compared the incidence of SIRS and several clinicalparameters among the groups. SIRS was diagnosed by thecriteria used by Boehme.^7^ According to these criteria, theexistence of two of the following symptoms was sufficient forthe diagnosis of SIRS: fever < 36°C or > 38°C, heart rate >90 beats/min, respiratory rate > 20 breaths/min or PaCO2 < 32mmHg, leukocytes < 4 000 cells/μl or > 12 000 cells/μl or > 10%polymorphonuclear leukocytes for at least 24 hours. All patientswere cooled to 32°C during cardiopulmonary bypass (CPB).

Patients who underwent emergent CABG or simultaneous valve/vascular surgery were excluded from the study. In addition, patients given postoperative anti-inflammatory drugs were also excluded from the study.

## Statistical analysis

All of the statistical tests were conducted using the Statistical Package for the Social Sciences for Windows version 22 (SPSS Inc, Chicago, IL, USA). Group comparisons of categorical data were assessed using Pearson’s chi-squared and Fisher’s exact tests and chi-squared trend analysis. Because the permanent variables did not have normal distributions (Kolmogorov–Smirnov test, p < 0.05), the Mann–Whitney U-test was used to compare the two groups, and the Kruskal–Wallis H-test (post hoc Bonferroni corrected Mann–Whitney U-test) was used to compare multiple groups. The associations between SIRS and other variables were evaluated using Spearman’s rho correlation analysis.

## Results

We found no statistically significant differences among the groups regarding gender, incidence of diabetes, chronic obstructive lung disease, chronic renal failure or prior open-heart surgery (p > 0.05). Smoking rates, pre-operative haemoglobin levels, and body surface area were significantly lower in group 3 than in the other groups, and the incidence of hypertension was significantly lower in group 1 compared to the other groups (p < 0.05). The average EuroSCORE value was higher in group 3 than in the other groups (p < 0.05; Table 1).

**Table 1:** Demographic data

*Parameters*	*Group 1(n = 61) n (%)*	*Group 2 (n = 83) n (%)*	*Group 3 (n = 85) n (%)*	*p-value*
Age (years)	36.7	61.3	77.9	
Male gender	49 (80.3)	63 (75.9)	55 (64.7)	0.083
Diabetes mellitus	14 (23)	36 (43.4)	21 (24.7)	0.937
COPD	1 (1.6)	3 (3.6)	6 (7.1)	0.107
Hypertension	17 (27.9)	49 (59)	54 (63.5)	0.000
CRF	4 (6.6)	3 (3.6)	1 (1.2)	0.082
Smoking	35 (57.4)	42 (50.6)	24 (28.2)	0.001
Redo surgery	0	0	2 (2.4)	0.107
EuroSCORE	1.95 ± 2.07	3.69 ± 2.24	5.2 ± 1.7	0.000
Pre-operative Hb	13.02 ± 1.89	12.57 ± 1.71	11.93 ± 1.49	0.000
BSA	1.88 ± 0.18	1.77 ± 0.16	1.68 ± 0.16	0.000

COPD: chronic obstructive pulmonary disease, CRF: chronic renal failure, Hb: haemoglobin, BSA: body surface area.

Comparisons of off-pump bypass surgery rates, CPB time, cross-clamping time, intra-aortic balloon pump use and revision ratios revealed no statistically significant differences among the groups (p > 0.05). However, the amount of postoperative drainage and peri-operative blood transfusions were significantly hign her in group 2 than ithe other groups (p < 0.05; Table 2).

**Table 2:** Peri-operative data

*Parameters*	*Group 1(n = 61) n (%)*	*Group 2(n = 83) n (%)*	*Group 3(n = 85) n (%)*	*p-value*
Off-pump CABG	15 (24.6)	20 (24.1)	19 (22.4)	0.745
CPB time (min)	82.21 ± 49.45	88.05 ± 29.05	83.58 ± 29.76	0.105
Cross-clamping time (min)	45.9 ± 30.62	46.53 ± 19.17	43.72 ± 18.78	0.536
Revision	5 (8.2)	8 (9.6)	11 (12.9)	0,343
Drainage (ml)	581 ± 294	480 ± 268	670 ± 501	0.004
Blood transfusion (IU)	2.25 ± 1.45	1.7 ± 0.95	3.26 ± 3.38	0.045
IABP	8 (13.1)	6 (7.2)	15 (17.6)	0.321

CABG: coronary artery bypass graft, CPB: cardiopulmonary bypass, IABP: intra-aortic balloon pump.

We found no significant differences in length of intensive care unit or hospital stay, incidence of neurological complications, and mortality rates among the groups (p > 0.05). However, the incidence of SIRS was significantly higher in group 3 than in group 1 (p < 0.05; Table 3), and the weaning period was significantly shorter in group 1 than in the other groups (p < 0.05).

**Table 3:** Postoperative data

*Parameters*	*Group 1 (n = 61) n (%)*	*Group 2 (n = 83) n (%)*	*Group 3 (n = 85) n (%)*	*p-value*
Weaning period (h)	10.67 ± 7.55	13.9 ± 10.01	14.28 ± 10.25	0.000
ICU stay (day)	2.92 ± 1.45	3.2 ± 2.69	3.52 ± 2.42	0.346
Hospital stay (day)	7.41 ± 3.96	8.59 ± 12.22	7.32 ± 3.12	0.736
Neurological complications	1 (1.6)	2 (2.4)	2 (2.4)	0.786
Mortality	3 (4.9)	5 (6.0)	12 (14.1)	0.083
SIRS	42 (68.9)	70 (84.3)	78 (91.8)	0.000

ICU: intensive care unit, SIRS: systemic inflammatory response syndrome.

Analysis of the predictive factors for SIRS revealed a statistically significant but weak positive correlation of SIRS with age, EuroSCORE value, on-pump CABG and intra-aortic balloon pump use. By contrast, we found a statistically significant but weak negative correlation of SIRS with pre-operative haemoglobin levels and off-pump CABG (p < 0.05). No other statistically significant relationships were found between SIRS and the other variables (p > 0.05; Table 4).

**Table 4:** Predictive factors for SIRS following CABG

	SIRS	
*Parameters*	*r*	*p-value*
Age	0.254	0.000
Diabetes mellitus	0.103	0.121
COPD	–0.017	0.799
Smoking	0.028	0.672
CRF	–0.040	0.544
Hypertension	0.103	0.119
Redo surgery	–0.082	0.216
EuroSCORE	0.179	0.007
Ejection fraction	–0.037	0.580
Neurological complications	0.068	0.308
Hospital stay	–0.015	0.837
Pre-operative haemoglobin level	–0.164	0.013
Body surface area	–0.073	0.272
Off-pump CABG	–0.186	0.005
On-pump CABG	0.208	0.002
CPB period	0.140	0.062
Cross-clamping period	0.138	0.065
Intra-aortic balloon pump use	0.138	0.037
Drainage amount	0.048	0.471
Blood transfusion	0.060	0.531
Revision	–0.035	0.602

COPD: chronic obstructive pulmonary disease, CRF: chronic renal failure, CABG: coronary artery bypass grafting, CPB: cardiopulmonary bypass, SIRS: systemic inflammatory response syndrome.

## Discussion

CPB itself may trigger systemic inflammation; however its role is controversial because inflammation may be induced by several factors other than CPB. Tissue damage, endotoxaemia and contact of blood with a non-endothelial surface during CBP are thought to trigger systemic inflammation during open-heart surgery,^8^,^9^ which may lead to SIRS.

The reported incidence of SIRS during the 24-hour postoperative period widely varies from 27 to 96%; this variability may be explained by the different diagnostic criteria, such as clinical parameters versus the measurement of pro-inflammatory mediators,^7^ used in the various studies. For instance, Sasse et al.^10^ found a SIRS incidence of 39% in paediatric patients with a history of prior cardiac surgery, whereas MacCallum et al.^11^ reported that the incidence of SIRS was 96.2% in an adult cardiothoracic intensive care unit. Our finding that the incidence of SIRS was 83% in all age groups is consistent with that of MacCallum et al.^11^

Given the wide range in age of the patients undergoing CABG, marked differences in postoperative outcomes have been observed in the different age groups. Several studies have reported widely varying results, particularly those including octogenarians. Therefore, although some studies have found a poor postoperative outcome in older compared to younger adult patients, others found that CABG was a safe procedure for octogenarian patients. For example, Sumin et al.^12^ assessed postoperative outcomes according to age in patients who underwent CABG. The authors found that the rates of hospital mortality and postoperative complications were significantly higher in patients older than 70 years compared to those younger than 60 years. Similarly, Wilson et al.^13^ reported that the rates of mortality and postoperative complications were higher in patients older than 75 years than in younger patients.

In a similar study, Arıtürk et al.^14^ reported that advanced age was a risk factor for 30-day mortality in patients who underwent CABG and mitral valve repair as a result of ischaemic mitral regurgitation. Moreover, an investigation of risk factors predicting neurological complications following CABG found that advanced age was a significant risk factor.^15^ Conversely, several investigators have reported that age had no effect on postoperative outcomes after CABG. In a study of 8 890 patients, Karimi et al.16 found that age was not a predictive factor for mortality. A meta-analysis of 12 697 older patients found that the CABG postoperative outcomes were satisfactory.^17^ An investigation of arterial graft use for CABG in patients older than 70 years found that CABG was safe and effective for older individuals.^18^

We found no significant differences in mortality or neurological complication rates among the age groups in our study. Our finding that the average EuroSCORE value was higher in older (group 3) than younger (groups 1 and 2) patients is noteworthy because high EuroSCORE values predict poor early and late postoperative outcomes.^19^

Aging is associated with increased inflammatory activity;20 however, the role of aging on the immune response to various stimuli is controversial. Krabbe et al.^20^ reviewed studies investigating the role of gene polymorphisms in inducing inflammation. Some studies found no association between age and systemic inflammatory mediators,^21^,^22^ whereas others reported a marked increase in inflammatory cytokines; in particular, interleukin-1 (IL1), IL6 and tumour necrosis factoralpha (TNF-α) levels were higher in older than in younger patients.^23^,^24^ We used clinical parameters but not markers of inflammation to evaluate the effects of age on SIRS. We found that the incidence of SIRS was significantly higher in patients older than 75 years than in those younger than 40 years. Few studies have used clinical parameters to investigate the correlation between age and SIRS in the postoperative period after CABG.

Previous investigations of predictive factors for SIRS have yielded important findings both in terms of identifying and preventing risk factors; however, it is surprising that so few studies have investigated the risk factors associated with SIRS in open-heart surgery, and of those, none have focused on CABG. A study investigating the correlation between intraoperative blood transfusion and SIRS found that intra-operative blood transfusion, low pre-operative functional capacity, liver dysfunction, chronic obstructive pulmonary disease, male gender, pre-operative steroid therapy, history of pre-operative haemodialysis and being older than 74 years were risk factors for postoperative SIRS.^25^,^26^ An investigation of SIRS in patients who had undergone transaortic valve implantation found that the predictive factors for SIRS were contrast amount, major bleeding, major vascular trauma and blood transfusion.^27^

A study of patients who underwent paediatric heart surgery found that predictive factors for SIRS were age, low body weight, and CBP and cross-clamping times.^8^ A similar study in a paediatric population found that CPB time, low body weight (< 10 kg) and right-to-left shunt were predictive factors for SIRS. Our findings that age, pre-operative haemoglobin levels, EuroSCORE value, on-pump CABG and intra-aortic balloon pump use were predictive factors for SIRS are consistent with those of previous studies. Our sample size was adequate; however, a limitation of our study is that pro-inflammatory mediators were not used to diagnose SIRS.

## Conclusion

We found that age was a risk factor for SIRS in patients undergoing CABG. For this reason, it should be borne in mind that the risk of developing SIRS in elderly patients increases, and accordingly, precautionary measures must be taken. Nevertheless, larger randomised clinical studies in patients undergoing CABG are needed to clarify the relationship between age and SIRS.
